# Anterior Cervical Discectomy and Fusion Complications and Thirty-Day Mortality and Morbidity

**DOI:** 10.7759/cureus.7643

**Published:** 2020-04-12

**Authors:** Sami Al Eissa, Faisal Konbaz, Sarah Aldeghaither, Monerah Annaim, Rayed Aljehani, Fahad Alhelal, Majed Abaalkhail, Ali A Alhandi

**Affiliations:** 1 Orthopaedics, King Abdulaziz Medical City, Ministry of National Guard Health Affairs, Riyadh, SAU; 2 Orthopaedics, King Saud Bin Abdulaziz University for Health Sciences, Riyadh, SAU

**Keywords:** acdf, spine, discectomy, fusion, saudi arabia

## Abstract

Background

Anterior cervical discectomy and fusion (ACDF) is a commonly used procedure. However, few studies reported post-operative complications. This study looks into the prevalence of possible complications and the mortality rate in the first 30 days postoperatively.

Methods

A retrospective review of patients who underwent ACDF for degenerative disc disease from 2008-2017, in a single center in Riyadh, Saudi Arabia was performed. Patient demographic data, comorbidities, operative notes, immediate and delayed complications were all collected, with a minimum of 30 days follow-up.

Results

Out of 434 medical charts reviewed, 163 met the inclusion criteria. Mean population age was 52 ± 11 years. Elective cases comprised 90% of sample and most patients had one or two levels operated on, 95% had ACDF and only 5% had corpectomy. The drain was left in 69% of patients and planned intensive care admission was done for 3%. Instrumentation and graft was used, with 92% needing a cage plus plate. Intraoperative complications were minimal. Mean hospital stay was 12.5 ±18 days. Majority of population had no complications in a 30 days period (98.2%). Only one case underwent revision surgery.

Conclusions

While ACDF is considered a safe procedure, postoperative complications may have long-term implications. This study showed minimal complications in the immediate postoperative period, but due to the limited sample size, a study with larger population is needed to further confirm the results.

## Introduction

Anterior cervical discectomy and fusion (ACDF) has been a commonly used surgical procedure that was described by Cloward and Robinson and Smith [1-2]. It is used to decompress the cervical spinal cord and the nerve roots in cases of herniated intervertebral disc, cervical radiculopathy, spondylosis, myelopathy or deformity caused by trauma, tumor or infection [3]. Although ACDF is considered a safe procedure and its complications are relatively rare and manageable, some complications may be serious and could last for several weeks to months. Intraoperative complications include the risk of recurrent laryngeal nerve injury; such injury rarely causes permanent post-operative hoarseness. Dysphagia is a relatively common complication and it usually lasts for days to months due to edema and pressure effect [3-8]. Other post-operative complications have also been reported, including soft tissue hematoma, respiratory problems, adjacent level disease, infection, epidural abscess, hardware failure, pseudarthrosis, and non-union [3-8]. There are risk factors that have been described in the literature linking ACDF related complications to patient’s age, female gender, smoking, type of hardware, having a revision surgery and multi-level surgery [9]. This study aimed to explore the mortality and morbidity rates in the first 30 days following ACDF procedure and to identify the associated risk factors in the Saudi population. Due to the lack of data concerning this population, this study offers a valuable addition to the literature.

## Materials and methods

A retrospective cohort study of 434 patients who underwent primary ACDF surgery for cervical radiculopathy and spondylosis between 2008-2017 with a minimum of 30 days follow up was performed. Orthopaedic surgery and neurosurgery patients from the same institute were included in the study. Patients aged bellow 20 or above 80, patients who had dysphagia and/or dysphonia preoperatively, patients who underwent combined anterior and posterior cervical spine surgeries during the same admission, patients who underwent cervical arthroplasty procedures, and patients who were treated for other indications other than degenerative pathology (e.g. Trauma, tumor, infection etc.) were excluded from the study. Patient demographics such as (age, gender, BMI, smoking, and other comorbidities) and operative notes (including blood loss, use of drain, type of graft, number of levels involved, duration of surgery, intraoperative complications, etc.) were documented. Thirty-day complication variables defined as return to ICU, revision surgery, pneumonia, hematoma, wound infection and death (Table 1). Incidence of persistent dysphagia and dysphonia (post 30 days) was also assessed.

**Table 1:** Thirty-day complication variables defined

	30 Days Complications		
	N	Frequency	%
Revision surgery	163	0	0
Return to ICU	163	1	0.6
Return to OR	163	1	0.6
Pneumonia	163	0	0
Hematoma	163	2	1.2
Wound infection	163	1	0.6
Death	163	0	0

Surgical procedure

After applying the standard general anesthesia technique, the patient was positioned in a supine position. A longitudinal or vertical surgical incision was made at the targeted level of decompression, followed by superficial dissections through the fascia and platysmal muscle, retraction of the esophagus medially and the sternocleidomastoid with the carotid sheath laterally. A deep dissection by splinting the longus colli muscles and anterior longitudinal ligament to expose the vertebral body was done. This was followed by decompression of the targeted level by removal of the affected disc level and osteophytes. The bone graft was then applied followed by plate and screws if planned beforehand.

Statistical analysis

The data was analyzed using Statistical Package for the Social Sciences (SPSS) (IBM Corp. Released 2013. IBM SPSS Statistics for Windows, Version 22.0. Armonk, NY: IBM Corp). Quantitative data from the patients’ charts such as age, length of stay, and BMI was included in descriptive statistics such as mean, standard deviation and frequencies. We used the non-parametric test for non-numerical data, and the students t-test for normal distribution data. A multivariant regression analysis was carried out to assess relationship between variables and possible complications.

## Results

Out of the 434 medical charts reviewed, 163 met the inclusion criteria for the 10-year period between 2008-2017; patients from both neurosurgery and the orthopedic surgery case pool were included. Mean population age was 52 ± 11 years. Female to male ratio was 42% to 58% and smokers comprised 22% of the population. Cardiac comorbidities were found in 28.8% of the patients, of which 2.4% were on anticoagulation. Diabetes and Hypertension prevalence in the sample were at 35.5% and 31% respectively (full descriptive statistics Table 2). ASA Physical Status Classification System was reviewed and mentioned in Table 3.

**Table 2:** Baseline characteristics

Variable		N (%)
Age (Mean, years)		51.57
Gender	Male	95 (58%)
	Female	68 (42%)
BMI (Mean, kg)		29.21
Smoking status	Smokers	36 (22%)
	Non-smokers	127 (78%)
Cardiac comorbidity		47 (28.8%)
HTN		58 (35.5%)
DM		51(31.2%)
COPD		2 (1.2%)
Bleeding disorders		0 (0%)
Use of anti-coagulant		4 (2.4%)
Pulmonary comorbidities (n) BA		9 (5.5%)
Recovered TB		1 (0.6%)
Previous PE		1 (0.6%)

**Table 3:** ASA Physical Status Classification System

ASA	Frequency	%
1	30	18.4
2	98	60.1
3	34	20.9
4	1	0.6

Operative statistics

Elective cases comprised 90% of the sample and most of the patients had one or two levels operated on (56% and 39% respectively), 95% had ACDF procedure performed and only 5% ended with a corpectomy (9 patients). One patient needed an intraoperative transfusion, drain was left in 69% of patients and planned intensive care admission was only done for 5 patients (3% of the sample). Neuromonitoring was used for 8 patients only (4.9%). Instrumentation and graft usage are detailed in Table 4 with 92% needing a cage plus plate configuration and 63% had an allograft used. Intraoperative complications were minimal mounting to one recurrent laryngeal nerve injury in one patient and vascular injury in another. Postoperative complications were also at low levels with 93.8% complication free at 30 days. One patient was noted to have a wound infection on day 8 post-operatively due to retropharyngeal abscess. Seven patients experienced dysphagia that took more than 30 days to resolve, five of which had concurrent dysphonia (Table 5). Mean hospital stay 12.5 ±18 days. A multivariant regression analysis did not note any significant relationship with length of stay and complication rate. Although length of hospital stay was significantly associated with older age, dyslipidemia, C3-spine levels instrumentation, use of bone graft and finally 500 cc or more intraoperative blood loss (Table 6).

**Table 4:** Instrumentation and graft usage

Instrumentation		Frequency	%
Cage		13	8
Cage + Plate		150	92
Grafted	Allograft	104	63.8
	Allograft + Autograft	4	2.5
None grafted		22	13.5

**Table 5:** Thirty-day Complication incidence in included sample

Patient	Complications			
1	Retro pharyngeal abscess			
2		Recurrent laryngeal nerve injury	Dysphagia	
3				Dysphonia
4			Dysphagia	Dysphonia
5				Dysphonia
6			Dysphagia	
7			Dysphagia	Dysphonia
8			Dysphagia	Dysphonia
9			Dysphagia	Dysphonia
10				Dysphonia

**Table 6:** Multivariant Regression Analysis denoting relationships of comorbidities, operative time, Number of levels operated, and blood loss with length of hospital stay. *correlation between length of hospital stay and the estimated risk factors *p value significant if <0.01. CVA=Cerebrovascular accident; HTN=Hypertension; DM=diabetes mellitus; COPD=Chronic obstructive pulmonary disease; DLP=Dyslipidemia; CATH= Cardiac catheterization

		Correlation		
		Coef.	SD	P value
Age		0.25	0.1	0.01
Gender		-0.44	2.22	0.84
BMI		0.121	0.15	0.43
Smoking		1.55	2.76	0.57
HTN		3.43	2.62	0.19
DM		-2.22	2.57	0.39
COPD		2.63	7.78	0.73
DLP		6.05	2.71	0.03
Cardiac comorbidities	CVA	3.77	5.75	0.51
	Heart disease	5.66	7.83	0.47
	Fibrillation	16.06	13.6	0.24
	CATH	2.19	7.8	0.78
	Operative time (hours)	3.56	2.55	0.16
Number of C-spine	2 levels	3.19	2.35	0.18
	3 levels	12.96	5.65	0.02
	4 levels	9.9	8.21	0.23
	Estimated Blood loss >500cc	20.42	8.04	0.01

## Discussion

This paper showed a minimal number of complications in the first 30 days postoperatively. In early postoperative period, 5.5% of the patients experienced dysphagia and/or dysphonia that did not resolve within the 30-day period. In the literature, dysphagia was one of the most common complications post ACDF surgery [10]. In a retrospective study in 2018, Mullins et al. reported that dysphagia is as minimal as 7 patients out of 1123 [10]. Fisahn et al. compared a stand-alone cage to a plate-cage construct with 8.9% of the population reported to experience chronic dysphagia. A predominance in that plate-cage group without clinical significance was noted in that study [11]. Wang et al. reported a higher number of dysphagia reaching 20% in the post-operative period [12]. Moreover, Riley et al. reported dysphagia rates up to 30% of their population, and it increased with the increase of number of levels instrumented [13]. In a prospective study, Lee et al. found that female revision surgery and multi-level surgery were associated with a higher rate of dysphasia [8]. Yadav et al. found that dysphagia was the most common post-operative complaint (16.4%) followed by neurological deterioration (7.9%); one patient suffered from subcutaneous emphysema and hemoptysis due to pharyngeal perforation [14].

Length of hospital stay is an important indicator of possible complications or associated morbidities to any surgical procedure. Khanna et al. studied the outcome of 6940 patients who underwent a single-level ACDF. The outcomes assessed included duration of hospital stay, 30-day medical and surgical complications, reoperation, readmission, and mortality. The results were that a total of 5162 patients had an inpatient hospital stay, whereas 1778 patients had outpatient surgery. Compared to outpatient surgery, the overall complication rate was higher in the inpatient arm including 30-day readmission rate. However, mortality was the same with 0.1% in both groups [15]. A less common complication such as esophageal perforation has been reported in some case reports. Park et al. reported a case of recurrent esophageal perforation after 20 and 25 years postoperatively due to screw pull out. The patient had spontaneous healing in the first perforation, however he required surgical repair the second time [16]. Phan et al. recommended close attention to anesthesia time as they found it increases the odds of complications post ACDF such as thromboembolic events, prolonged hospital stay, and the return to operation table [17]. Song et al. reviewed 785 patients who underwent ACDF for post-operative acute airway obstruction (AAO) secondary to retropharyngeal hematoma. Nine patients developed AAO (1.15%), with no significant risk factors identified [18]. Sagi et al. showed that prolonged procedures, more than 5 hours, blood loss more than 300 ml and more than three levels operated were significantly associated with airway complications [19]. 

Approximately 3% of our population had to go into revision surgery in the long term with only one case which fit within the 30-day scope of this paper (Table 6) [17]. No mortality was noted within the 30-day time period of this paper, although expanding the timeline showed one case of death due to pneumonia and subsequent acute respiratory distress syndrome (ARDS) within the first year post operatively. Kelly et al. in their retrospective review that included 50926 patients, seven and 55 patients had cardiopulmonary complications including MI and PE respectively, 24 patients experienced vertebral artery tear and mortality was noted in 93 patients in the sample [20]. This constitutes 0.1% of their sample which is not far from the 0.6% noted in this population. It is within reason to expect similar results given a larger sample size.

**Table 7:** Long-term revision rate in the sample – minimum of 2 year follow up

Patient #	Revision Timeline Post operatively	Reason
1	16 months	Dysphagia – plate removal
2	3 years	Adjacent level disease
3	6 months	Adjacent level disease
4	3 years	Adjacent level disease, Cord compression
5	8 days	Retropharyngeal abscess

We acknowledge the sample size of this study as a weakness, although it was expected for a single center study. Furthermore, the lack of reporting the disability index and subjective analysis is noted as a weakness and can be attributed to the retrospective nature of the study and the lack of complete documentation in the older paper-based records. We recommend a minimum of a 2- year follow up prospective multicenter study that addresses this population. Such a study will give valuable insights on the associated morbidity and mortality in the short and long term with the ACDF procedure. 

## Conclusions

While ACDF is considered a safe procedure, postoperative complications, when present, may have long-term ramifications. This paper reports a low complication rate in the first 30 days postoperatively and recommends prospective long-term studies. 

## References

[1] Smith GW, Robinson RA (1958). The treatment of certain cervical-spine disorders by anterior removal of the intervertebral disc and interbody fusion. J Bone Joint Surg Am.

[2] Cloward RB (1958). The anterior approach for removal of ruptured cervical disks. J Neurosurg.

[3] Steinmetz MP, Benzel EC (2011). Spine surgery 2-Vol set e-book: techniques, complication avoidance and management.

[4] Alturki A, Basamh M, Awwad W, Jarzem P (2014). Multi-corporal abscess formation due to esophageal perforation post anterior cervical discectomy and fusion (ACDF). Int J Neurol Neurosurg.

[5] Shriver MF, Lewis DH, Kshettry VR, Rosenbaum BP, Benzel EC, Mroz TE (2015). Pseudoarthrosis rates in anterior cervical discectomy and fusion: a meta-analysis. Spine J.

[6] Yalamanchili PK, Vives MJ, Chaudhary SB (2012). Cervical spondylotic myelopathy: factors in choosing the surgical approach. Adv Orthop.

[7] Wang Z, Zhou L, Lin B, Song K, Niu Q, Ren D, Tang J (2018). Risk factors for non-fusion segment disease after anterior cervical spondylosis surgery: a retrospective study with long-term follow-up of 171 patients. J Orthop Surg Res.

[8] Lee MJ, Bazaar R, Furey CG, Yoo J Risk factors for dysphagia after anterior cervical spine surgery: a two-year prospective cohort study. Spine J.

[9] Ji G, Oh C, Shin D, Ha Y, Kim K, Yoon D, Yudoyono F (2015). Stand-alone cervical cages versus anterior cervical plates in 2-level cervical anterior interbody fusion patients: analysis of adjacent segment degeneration. J Spinal Disord Tech.

[10] Mullins J, Pojskic M, Boop FA, Arnautovik KI (2018). Retrospective single-surgeon study of 1123 consecutive cases of anterior cervical discectomy and fusion: a comparison of clinical outcome parameters, complication rates, and costs between outpatient and inpatient surgery groups, with a literature review. J Neurosurg Spine.

[11] Fisahn C, Schmidt C, Rustagi T (2018). Comparison of chronic dysphagia in standalone versus conventional plate and cage fusion. World Neurosurg.

[12] Wang T, Ma L, Yang DL (2017). Factors predicting dysphagia after anterior cervical surgery: a multicenter retrospective study for 2 years of follow-up. Medicine (Baltimore).

[13] Riley LH III, Skolasky RL, Albert TJ, Vaccaro AR, Heller JG (2005). Dysphagia after anterior cervical decompression and fusion: prevalence and risk factors from a longitudinal cohort study. Spine (Phila Pa 1976).

[14] Yadav R, Chavali S, Chaturvedi A, Rath GP (2017). Post-operative complications in patients undergoing anterior cervical discectomy and fusion: a retrospective review. J Neuroanaesthesiol Critical Care.

[15] Khanna R, Kim RB, Lam SK, Cybulski GR, Smith ZA, Dahdaleh NS (2018). Comparing short-term complications of inpatient versus outpatient single-level anterior cervical discectomy and fusion: an analysis of 6940 patients using the acs-nsqip database. Clin Spine Surg.

[16] Park MK, Cho DC, Bang WS, Kim KT, Sung JK (2018). Recurrent esophageal perforation after anterior cervical spine surgery: case report. Eur Spine J.

[17] Phan K, Kim JS, Kim JH, Somani S, Di'Capua J, Dowdell JE, Cho SK (2017). Anesthesia duration as an independent risk factor for early postoperative complications in adults undergoing elective ACDF. Global Spine J.

[18] Song KJ, Byung WC, Lee DH, Lim DJ, Oh SY, Kim SS (2017). Acute airway obstruction due to postoperative retropharyngeal hematoma after anterior cervical fusion: a retrospective analysis. J Orthop Surg Res.

[19] Sagi Sagi, HC HC, Beutler W, Carroll E, Connolly PJ (2002). Airway complications associated with surgery on the anterior cervical spine. Spine (Phila Pa 1976).

[20] Kelly MP, Eliasberg CD, Ajiboye RM, McAnany SJ, SooHoo NF (2018). Reoperation and complications after anterior cervical discectomy and fusion versus cervical disc arthroplasty: a study of 52,395 cases. Eur Spine J.

